# Reconfiguration of the cortical-hippocampal interaction may compensate for Sharp-Wave Ripple deficits in APP/PS1 mice and support spatial memory formation

**DOI:** 10.1371/journal.pone.0243767

**Published:** 2020-12-31

**Authors:** Bartosz Jura, Dariusz Młoźniak, Hanna Goszczyńska, Katarzyna Blinowska, Nathalie Biendon, Nathalie Macrez, Pierre Meyrand, Tiaza Bem

**Affiliations:** 1 Nałęcz Institute of Biocybernetics and Biomedical Engineering, Polish Academy of Sciences, Warsaw, Poland; 2 Department of Biomedical Physics, Faculty of Physics, University of Warsaw, Warsaw, Poland; 3 Institut des Maladies Neurodégénératives, Université de Bordeaux, UMR 5293, Bordeaux, France; 4 CNRS, Institut des Maladies Neurodégénératives, UMR 5293, Bordeaux, France; 5 INSERM, Neurocentre Magendie, Bordeaux, France; Nathan S Kline Institute, UNITED STATES

## Abstract

Hippocampal-cortical dialogue, during which hippocampal ripple oscillations support information transfer, is necessary for long-term consolidation of spatial memories. Whereas a vast amount of work has been carried out to understand the cellular and molecular mechanisms involved in the impairments of memory formation in Alzheimer’s disease (AD), far less work has been accomplished to understand these memory deficiencies at the network-level interaction that may underlie memory processing. We recently demonstrated that freely moving 8 to 9-month-old APP/PS1 mice, a model of AD, are able to learn a spatial reference memory task despite a major deficit in Sharp-Wave Ripples (SWRs), the integrity of which is considered to be crucial for spatial memory formation. In order to test whether reconfiguration of hippocampal-cortical dialogue could be responsible for the maintenance of this ability for memory formation, we undertook a study to identify causal relations between hippocampal and cortical circuits in epochs when SWRs are generated in hippocampus. We analyzed the data set obtained from multielectrode intracranial recording of transgenic and wild-type mice undergoing consolidation of spatial memory reported in our previous study. We applied Directed Transfer Function, a connectivity measure based on Granger causality, in order to determine effective coupling between distributed circuits which express oscillatory activity in multiple frequency bands. Our results showed that hippocampal-cortical coupling in epochs containing SWRs was expressed in the two frequency ranges corresponding to ripple (130–180 Hz) and slow gamma (20–60 Hz) band. The general features of connectivity patterns were similar in the 8 to 9-month-old APP/PS1 and wild-type animals except that the coupling in the slow gamma range was stronger and spread to more cortical sites in APP/PS1 mice than in the wild-type group. During the occurrence of SWRs, the strength of effective coupling from the cortex to hippocampus (CA1) in the ripple band undergoes sharp increase, involving cortical areas that were different in the two groups of animals. In the wild-type group, retrosplenial cortex and posterior cingulate cortex interacted with the hippocampus most strongly, whereas in the APP/PS1 group more anterior structures interacted with the hippocampus, that is, anterior cingulate cortex and prefrontal cortex. This reconfiguration of cortical-hippocampal interaction pattern may be an adaptive mechanism responsible for supporting spatial memory consolidation in AD mice model.

## Introduction

Since the pioneering work of Alois Alzheimer in 1906, a substantial body of work has shown how Alzheimer’s disease (AD) alters cognitive functions such as working memory and semantic and episodic memories formation [1, 2]. Due to the high incidence of chronic disabilities that follow AD, rehabilitation therapy has become increasingly important in AD patient recovery. Over the last decade, research has primarily focused on deciphering the cellular and molecular mechanisms involved in AD memory impairments. Memory impairment in AD has been associated with the presence of soluble amyloid-β proteins (Aβ), which alter cellular and synaptic physiology in different parts of the brain [3–6]. Accumulation of Aβ has been extensively studied in transgenic APP/PS1 mice that overexpress mutant human genes for amyloid precursor protein (APP) and presenilin1 (PS1) [7] and develop memory deficits correlated with Aβ deposition [8]. In contrast, the physiopathology of the networks involved in memory processing has received far less consideration.

According to the two-stage model of spatial memory encoding, newly acquired information is first stored in the hippocampus and then transferred and consolidated in distributed networks of the neocortex, especially during quiescent, ‘off-line’ brain states, such as slow-wave sleep (SWS) [9, 10]. Sharp-Wave Ripples (SWRs), high frequency network events generated in the hippocampus, play a crucial role in memory consolidation and transfer of information to the cortex [11, 12]. Indeed, during SWRs synchronized memory trace reactivation occurs in the hippocampus and multiple cortical structures [11, 13]. SWRs occurrence is synchronized with cortical spindles and delta oscillations allowing a dialogue between the hippocampus and cortex [14, 15], and the experimental alteration of this dialogue impairs memory formation [16, 17]. Moreover, as recently demonstrated, reduced SWRs-delta wave coupling may be responsible for spatial learning impairments in 3xTg-AD mice, a triple-transgenic AD model expressing mutant APP, presenilin and Tau [18].

In addition, a relationship between SWRs and whole brain activity was demonstrated by measuring BOLD signals in macaque monkeys; SWRs-associated periods of subcortical silence, when neocortex and hippocampus were exclusively active, were revealed, which may allow for effective information processing within neocortical sites and hippocampus, as most energetic resources are directed to these brain areas [19]. Recently, an fMRI study of functional connectivity in humans showed bidirectional interactions between hippocampus and cortical sites during, and in close temporal proximity to, SWRs [20]. Moreover, investigation of firing patterns revealed a loop of neural activity propagating between auditory cortex and hippocampus [21]. In general, many studies have shown that neuronal activation or deactivation in multiple cortical structures is coordinated with the occurrence of SWRs (for review see [12]). However, it is difficult to visualize a clear-cut pattern of activity flow from the available data as the recordings are typically made at a single cortical site. Additionally, the directionality of the interaction remains unclear.

Hippocampal-cortical interaction in AD has not been thoroughly examined. We recently showed that 8 to 9-month-old APP/PS1 mice were able to learn a spatial reference memory task despite displaying a significant SWRs deficit [22]. This finding suggests that neural circuits involved in spatial memory formation, and the dialogue between cortex and hippocampus, may undergo an adaptive reconfiguration in APP/PS1 mice. To test this hypothesis, we undertook the study of hippocampal-cortical interaction in APP/PS1 and wild-type (WT) mice undergoing spatial memory consolidation. We analyzed the local field potential (LFP) in the hippocampus and in multiple cortical sites during SWS epochs containing SWRs on the data set obtained using intra-cranially recorded signals described in Jura et al, 2019. Our study was specifically interested in identifying the causal relationships between the hippocampus and cortex. We applied Directed Transfer Function (DTF), a connectivity measure based on Granger causality [23], to determine the magnitudes of effective coupling between remote neuronal circuits that are involved in the generation of signals in a given frequency band; we evaluated if and how this coupling changes during the occurrence of SWRs.

We report that hippocampal-cortical coupling occurs primarily in two frequency ranges, corresponding to SWRs and slow gamma band. In aged APP/PS1 mice (8–9 months old), a model of Alzheimer’s disease, the effective coupling between hippocampus and cortex during the occurrence of SWRs involves different cortical areas than in WT mice. These findings suggest a remodeling of the interaction occurred in APP/PS1 mice, which preserved the ability of memory consolidation.

## Materials and methods

### Experimental groups and surgical procedure

The APP/PS1 mice were progeny of crossbred male APP mice (Tg2576 mice bearing the APP Swedish mutation from Taconic Inc) and female PS1 mice (PSEN1dE9 from Jackson laboratory). A complete description of the line’s genetic background is provided in Jura et al, 2019. These mice have been used in multiple studies [24–26]. All mice were heterozygous for each transgene. Data were collected from 9 APP/PS1^+^ and 9 wild-type (WT) littermate controls (females, 8–9 months old). This time window was selected because amyloidosis and cognitive impairment were shown to begin at 5 months of age, and were clearly expressed at 8–10 months, in this double transgenic AD mouse model [24–26]. The Bordeaux University animal facility provided the mice whose genotype was performed by the genotyping facility of Neurocentre Magendie. They were housed one per cage in temperature- (22 ± 1°C) and humidity-controlled (50 ± 10%) conditions. Light was turned on at 7 am and maintained for 12 hours. Mice had *ad libitum* access to food and water prior to the experimental procedure.

For the implantation of multiple microelectrodes, we followed the same procedure as described in [22]. Briefly, microelectrodes were implanted using stereotaxic coordinates [27] into: the prefrontal cortex (PFC) (AP: +2.0 mm, L: −0.36 mm, V: −1.6 mm), anterior cingulate cortex (ACC) (AP: +0.98 mm, L: −0.32 mm, V: −1.48 mm), posterior cingulate cortex (PCC) (AP: −2.0 mm, L: −0.3 mm, V: −0.8 mm), retrosplenial cortex (RSC) (AP: −3.0 mm, L: −0.5 mm, V: −0.8 mm) and the CA1 region of left hippocampus (AP: −2.0 mm, L: −1.5 mm, V: −1.05 mm). The neck muscle activity was monitored using an electrode inserted into the neck muscles. Electrophysiological recordings and behavioral sessions were carried out 3–4 weeks after surgery.

The experimental procedures complied with official European Guidelines for the care and use of laboratory animals (directive 2010/63/UE) and were approved by the ethical committee of the University of Bordeaux (protocols A50120159 and 21377).

### Histological study of Aβ plaques

Independent samples from APP/PS1 (n = 3) and WT (n = 2) 8 to 9-month-old female mice were stained by immunofluorescence using a combination of primary antibodies (mouse anti-human Aβ (6E10) and rabbit anti-mouse Aβ (poly18058)) revealed by A488-conjugated anti-rabbit- and anti-mouse- secondary antibodies.

Mice were euthanized by cervical dislocation and transcardially perfused with cold NaCl 0.9%. After extraction, half of each brain was prepared for 40μm thick coronal sections by immersion-fixation in PFA (4%) overnight, cryopreservation, congelation and cryostat slicing. Slices were rinsed in phosphate buffered solution (PBS), then incubated in blocking buffer for 30 min and then incubated with primary antibodies (6E10 and poly18058, Ozyme, overnight at 4°C) diluted 1/1000 in PBS, goat serum 2%, triton 0.2%, BSA 0.1%. After washing, slices were incubated with secondary antibodies (goat anti-mouse A488 and goat anti-rabbit A488, Life Technologies; 1/500; 2h00 at room temperature), rinsed in PBS, nuclear counterstained with DAPI, mounted in Fluoromount and imaged with a Nanozoomer (Hammamatsu, x20 objective) at the Bordeaux Imaging Center (France-BioImaging). For each brain, 4 slices were analyzed for ACC and CA1, 3 slices for PFC and PCC and 2 slices for RSC. Plaques were analyzed with ImageJ; after thresholding the images, the regions of interest were drawn allowing for measurements of each region and particle analyses inside the regions of interest. The number of plaques was normalized to the region’s area measurement.

### Experimental procedure

Before starting spatial memory experiments mice were gradually food restricted to maintain their body weight at 85% of their baseline level throughout the experiments, while) free access to water was allowed. All procedures took place during the light cycle. For the memory test, an eight-arm radial maze was used as described in [22]. During each day of a two-day habituation mice first remained in their home cage for 90 min connected to the recording system, then they were placed into the maze in which all arms were baited with food rewards and left in the maze until all eight baited arms were visited and at least one food reward was consumed. Thereafter, the animals were placed back into their home cage for 90 minutes, again connected to the recording system. During learning the same procedure was applied during six consecutive days; however, the food rewards were distributed only in 3 selected arms of the maze. Each animal performed six trials per day, with each trial ending when all the rewards were eaten.

### Data acquisition

Recordings were performed in animals’ home cages immediately completing a session of the described hippocampus-dependent spatial learning task in an 8-arm radial maze. Electrophysiological activity of the brain was recorded with a Plexon neural data acquisition system (Omniplex, Plexon Inc). Neural data was sampled at 40 KHz and stored on a computer disk. Video signals were recorded in the animals’ home cages simultaneously with electrophysiological recording. We followed the procedure for identifying brain states and SWRs as described in [22]. In order to exclude the possibility that pathological seizure in APP/PS1 mice [28] altered the analysis, we assessed for the occurrence of such events. Among 9 APP/PS1 animals 6 were found to have expressed short lasting seizures of the duration ranging from 390 to 640 ms, with the frequency ranging from 4,32 to 21,96 events per hour. All of the time windows containing these seizures were removed from the analysis.

### DTF

Directed Transfer Function (DTF) is based on a Multivariate Autoregressive Model (MVAR):

For a vector of signals:

$$X(t)={\left(X_{1}(t),X_{2}(t),\ldots ,X_{k}(t)\right)}^{\text{T}}$$ (1)

we can express the MVAR model in the form:

$$X(t)=\sum_{j=1}^{p}A(j)X(t-j)+E(t)$$ (2)

where every **A**(*j*) is a *k*×*k* matrix of model coefficients, **E**(*t*) is a *k*-size vector of white noises, *p*–model order (the number of samples we take into account in regression). The MVAR model assumes that *X*_*i*_(*t*_*l*_)–a sample of signal *X*_*i*_(*t*) at a time *t*_*l*_−may be expressed as a sum of *p* previous values of signal *X*_*i*_(*t*) and also other signals of the **X(***t***)** vector, weighted by the model coefficients of matrices **A** plus a random noise vector **E**(*t*). Granger causality states that, for two time series, if the variance of the prediction error for the second time series is reduced by including past measurements from the first time series in the linear regression model, then the first time series can be said to cause the second time series. In a multichannel case, past measurements from all other channels from the set are included in the prediction. The MVAR model expresses the Granger causality principle [29] for an arbitrary number of channels. The transfer matrix of the MVAR defined in the frequency domain can be found from the model coefficients:

$$H(f)=\left|\sum_{m=0}^{p}A(m)\exp(-2\pi imf\Delta t)\right|.$$ (3)

where index *m* stands for the number of MVAR coefficients.

DTF is defined in terms of *H(f)*, which contains information about frequency characteristics and relations between signals:

$${\text{DTF}}_{ji}(f)=\frac{{\left|H_{ji}(f)\right|}^{2}}{\sum_{m=1}^{k}{\left|H_{im}(f)\right|}^{2}}.$$ (4)

*DTF*_*ji*_ *(f)* describes causal influence of channel *j* on channel *i* at frequency *f*. The estimator is proportional to the coupling between the time series [29], so by means of DTF, we can find the coupling strength in a given frequency band. DTF defined by formula (4) shows not only direct but also cascade coupling. For example, when following relationships between channels 1, 2, 3: 1: →2 and 2: →3 it will show also 1: →3 relation. To avoid this effect, direct DTF (dDTF) was introduced [30].

dDTF(f)=(DTF(f))(partialcoherence(f)) (5)

Partial coherence C_ji_(f) is defined by the means of minors of spectral matrix S(f), in the following way:

$$C_{ij}(f)=\frac{M_{ij}(f)}{\sqrt{M_{ii}(f)M_{jj}(f)}}$$ (6)

where **M**_ij_(f) is a minor of S(f) with the *i*th row and *j*th column removed. C_ji_(f) is nonzero only when the given relationship between channels is direct.

### Data processing

Automatic signal analysis was performed using custom-written Matlab (Mathworks, Inc., Natick, Massachusetts, United States) scripts. Signal filtering was performed using Matlab ‘cheby2’ filter and ‘filtfilt’ function in order to prevent any filter-introduced phase distortions that could undermine the DTF analysis. Behavioral states of wakefulness, REM sleep and SWS were scored manually by an experimenter using LFP signals, EMG and video recordings. Identified SWS bouts were used for further analysis. SWS was considered to be periods of behavioral immobility, as assessed using video recordings, with high-amplitude activity in the delta band (0.5–3 Hz) and low theta activity (4–10 Hz) and absent or weak tonic muscle activity.

Wideband signals were first downsampled to 500 Hz using the Matlab procedure ‘decimate’. Episodes of SWRs were detected automatically by thresholding the absolute value of the analytic form of the signals, obtained using Hilbert transform, taken from the CA1 channel, filtered first in the 100–250 Hz frequency band. The signal envelope found in this way was then z-scored, and periods in which it crossed 2 standard deviations (SD) and reached 5 SD of reference signal values were considered to be ripple events. Events separated by less than 20 ms were merged and events longer than 100 ms were discarded.

### Statistical analysis

All statistical analyses were performed in Matlab (MathWorks). Results are expressed as mean ± SEM. The statistical tests used were non-parametric since the distributions of resulting values were not normal. The significance level was set at p < 0.05. In the case of multiple comparisons, Bonferroni correction was applied. The data were collected from 9 APP/PS1 and 9 WT adult female mice.

## Results

Recordings were performed during 6 days of learning, in the home cage during the 90 min immediately before and after the learning sessions in which the animals learned the position of 3 baited arms in the 8-arm maze. It should be noted that although the 8 to 9-month-old APP/PS1 mice expressed two times more errors per trial than the WT group, they did not show a deficit in reference memory [22]. Surprisingly, though such memory relies on integrity of SWRs [16], we found a major impairment of the occurrence rate, oscillation frequency and learning-dependent dynamics of these network events in the APP/PS1 mice [22]. Since these findings suggested a reconfiguration of networks involved in SWRs-dependent memory formation, we applied dDTF, a connectivity measure based on Granger causality, in order to compare the interaction of hippocampal and cortical circuits in epochs containing SWRs in the WT and APP/PS1 mice.

**Fig 1:**
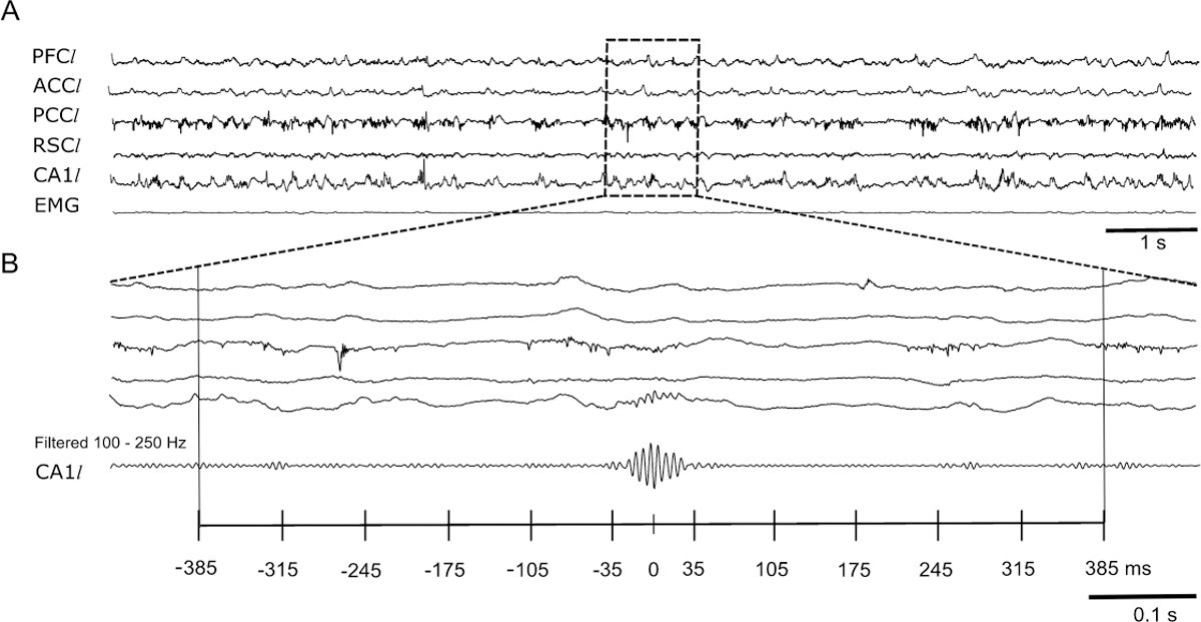
Representative example of an epoch containing SWR. (A) Simultaneous recordings of LFPs from left prefrontal cortex (PFC*l*), left anterior cingulate cortex (ACC*l*), left post cingulate cortex (PCC*l*), left retrosplenial cortex (RSC*l*), left dorsal hippocampus CA1 (CA1*l*), and an electromyogram of the neck muscle (EMG) during SWS (B) Enlargement of 1 sec recordings of the LFPs showing SWR oscillation within a 770 ms epoch in raw and filtered data. Notice a single SWR centered in the middle window and no other SWRs occurring before or after it (bottom panel).

We recorded the extracellular field potential in the left CA1 area of the hippocampus along with left PFC, ACC, PCC and RSC. These brain structures were chosen since they are involved in recall of recent and remote spatial memories [9]. Moreover, to determine more accurately the behavioral state of the animal (awake, SWS and REM sleep) the EMG of the neck muscle was recorded (Fig 1A). As previously reported, no differences were found in the occurrence of sleep episodes in WT and APP/PS1 group [22].

We focused on the analysis of 770 msec signal epochs containing a single SWR. Each epoch was divided into 11 time windows of equal length, with the middle window centered at the time point of the SWR’s peak amplitude (Fig 1B). For each consecutive window within the epoch the MVAR model was fitted to cortical (PFC, ACC, PCC, RSC) and hippocampal (CA1) signals. Then dDTF, a measure of effective coupling, was estimated according to Eq (5) for each time window. As the SWR’s duration was typically not longer than 70 ms, the middle window contained the full SWR, whereas adjacent windows corresponded to periods just before and after SWR. This allowed us to analyze the changes of effective connectivity related to the SWR’s occurrence. The data presented in this paper correspond to the first day of learning, after the learning session. Altogether n = 1,463 signal epochs that were analyzed in the WT group and n = 1,143 epochs for the APP/PS1 group.

### Effective coupling between hippocampus and neocortex is expressed in two frequency bands

**Fig 2:**
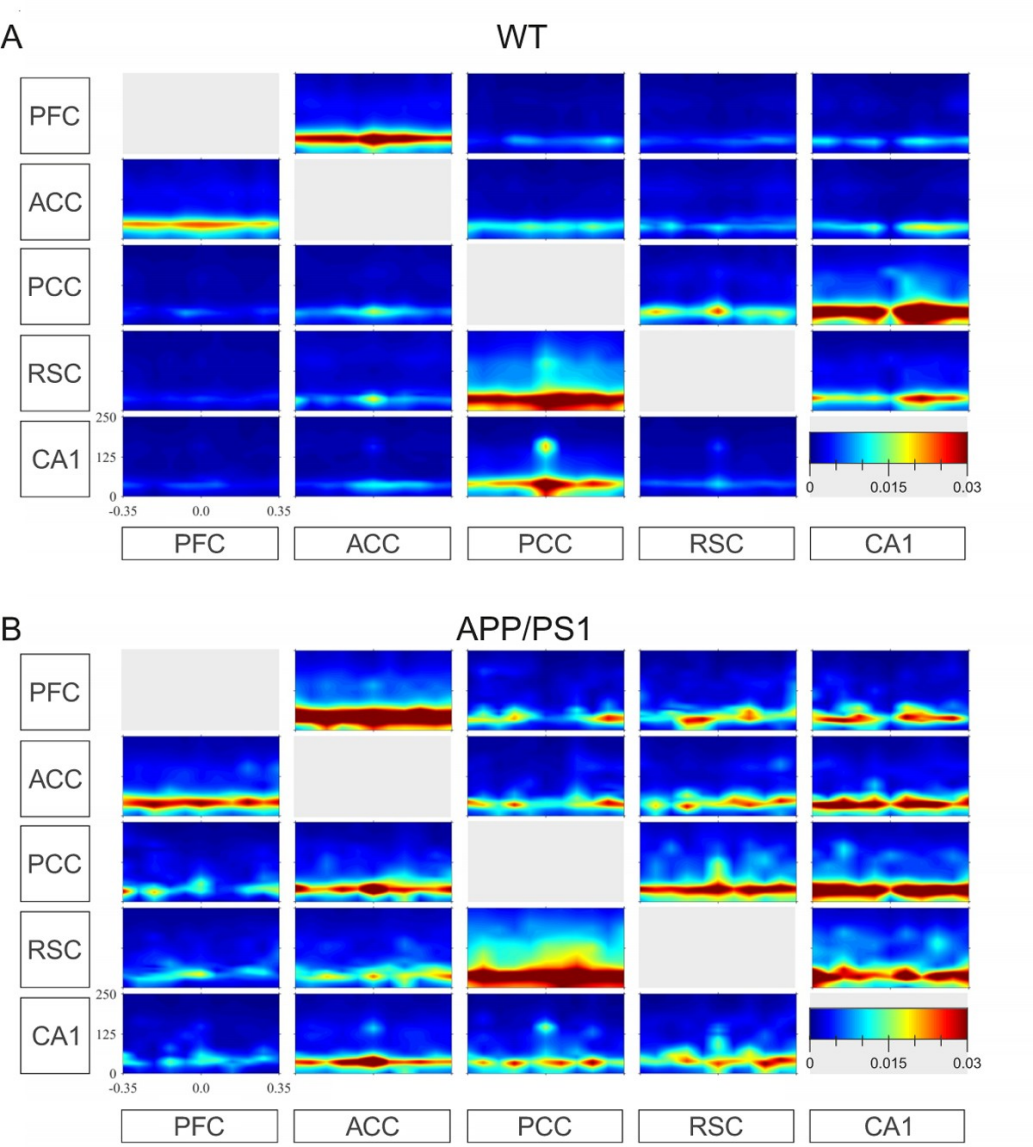
Time-frequency map of the effective coupling between CA1 and multiple cortical sites. dDTF plotted as function of time and frequency are averaged over WT (A) and APP/PS1 (B) groups. Every box shows effective coupling from the channel indicated below a given column to the channel indicated next to a given row. Time 0 indicates SWRs peak power. The color code indicates the value of the $\sqrt{\text{dDTF}}$. Note strong coupling in the SWRs (130–180 Hz) and slow gamma (20–60 Hz) bands. Units: seconds (x-axis) and Hz (y-axis).

Our analysis revealed the hippocampal-cortical effective coupling pattern was generally similar in both groups of animals. Fig 2 shows time-frequency maps of dDTF, averaged over the WT and APP/PS1 groups (Fig 2A and 2B, respectively). Each panel shows the value of the coupling between the channel labeled below a given column to the channel labeled next to a given row.

In both groups, the strongest coupling was found in the slow gamma frequency band (20–60 Hz) (see color code, Fig 2) and general features of the connectivity pattern, namely reciprocal connections between ACC and PFC, PCC and CA1 as well as between PCC and RSC, were similar. However, in the APP/PS1 animals additional coupling from ACC to CA1 and PCC and from CA1 to ACC and PFC was present. Besides the wide-spread coupling in the slow gamma frequency, a clear-cut, although weaker interaction, was identified in the SWR frequency band, mainly from PCC to CA1 (see 140–160 Hz in WT group, Fig 2A; 130–150 Hz in APP/PS1 group, Fig 2B).

Interestingly, in both slow gamma and SWRs frequency bands a modulation of the coupling strength was apparent for some pairs of channels at the very moment of the SWRs occurrence (see time point 0, Fig 2). In order to better assess the statistical significance of these changes, dDTF was integrated within each of the 11 time windows for each pair of channels and each animal, in the two frequency bands under consideration.

### Hippocampal-cortical coupling in the slow gamma band

**Fig 3:**
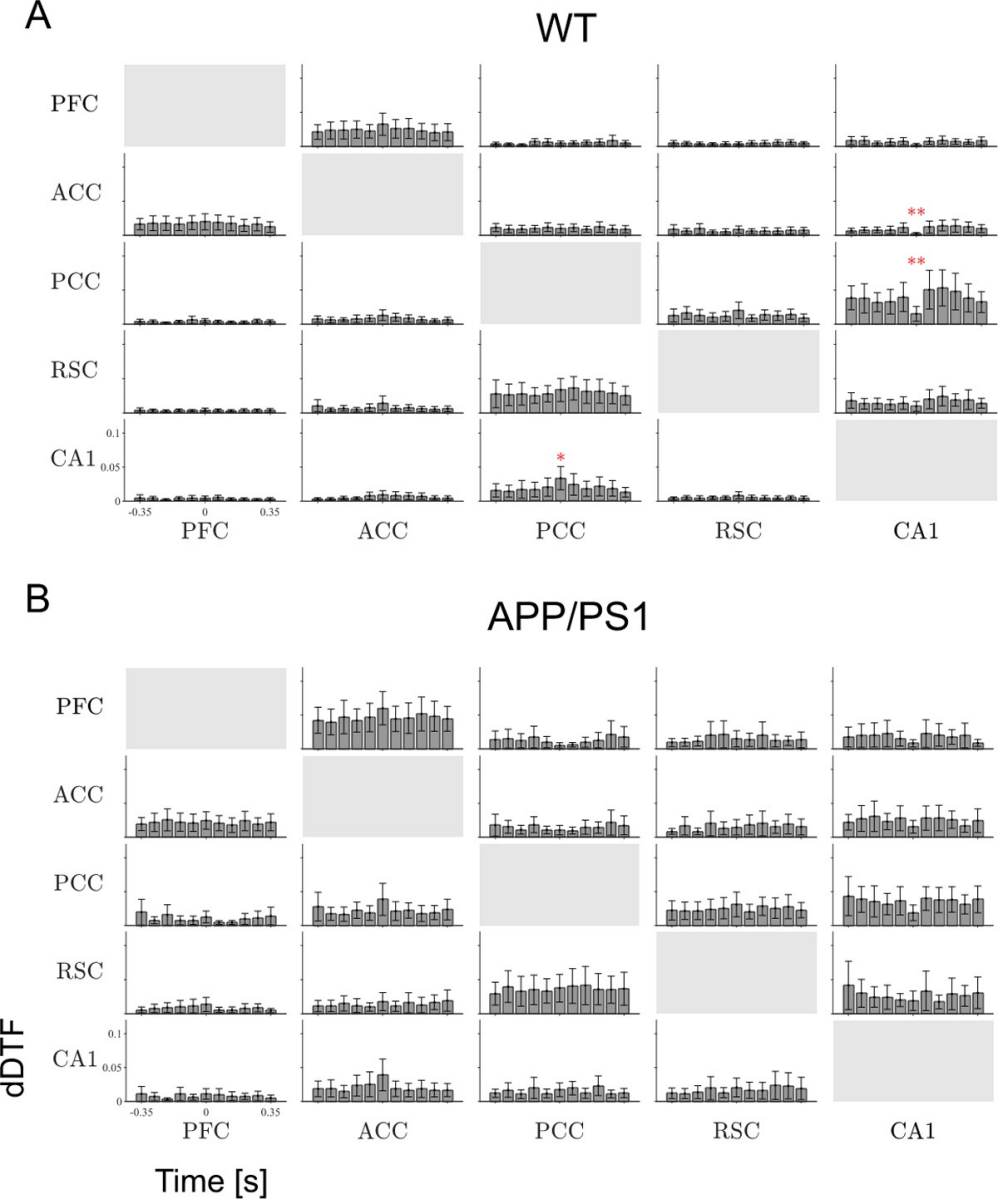
Effective coupling in the slow gamma band. Time course of dDTF value in the slow gamma (20–60 Hz) band, averaged over WT (A) and APP/PS1 (B) mice. Every box shows mean values of $\sqrt{\text{dDTF}}$ in the slow gamma frequency interval in consecutive time windows. Significant modification of the coupling value compared to the value of the coupling in the first bin is indicated by stars (Wilcoxon’s test with Bonferroni correction). Other abbreviations as in Fig 2. * p < 0.05, ** p < 0.01, *** p < 0.001. Time bin size is 70 ms.

Mean values of dDTF in the slow gamma frequency band, averaged over WT and APP/PS1 group for each of the time windows, are shown in Fig 3A and 3B, respectively. For most of the channel pairs the coupling was continuous, i.e., expressed during the entire epoch containing a SWR. In the WT group two loops of strong interaction were identified—first, between PCC, RSC and CA1 and, another, between ACC and PFC (Fig 3A). This is summarized in the schematic form in Fig 5A1 (see blue arrows, left panel). Interestingly, the strength of effective coupling from PCC to CA1 increased significantly at the precise time of SWRs emergence (N = 9, p = 0.0028, Wilcoxon’s test with Bonferroni correction) (see asterisk, Fig 3A and red arrow, Fig 5A2). During the same time window, the effective coupling from CA1 to PCC and from CA1 to ACC significantly decreased (N = 9, p = 0.0008 and p = 0.0005, respectively, Wilcoxon’s test with Bonferroni correction), (see asterisk, Fig 3A).

In the APP/PS1 animals the main pathways of hippocampal-cortical effective coupling, as expressed in the WT group, were still active (Figs 3B and 5A1). In addition, the strength of the coupling, averaged over the entire epochs containing SWR, increased compared to the WT group in following pathways: from ACC to PFC (N = 9, p = 0.0001, Wilcoxon’s test), between ACC and CA1 (N = 9, ACC to CA1: p = 0.006; CA1 to ACC: p = 0.013, Wilcoxon’s test), from ACC to PCC (N = 9, p = 0.0003, Wilcoxon’s test) as well as from RSC to PFC (N = 9, p = 0.022, Wilcoxon’s test). The dominant interaction pattern identified in the APP/PS1 group is schematically presented in Fig 5A (see blue arrows). Importantly, no SWRs-dependent modulation of coupling in the slow gamma frequency was found in the APP/PS1 mice.

In an attempt to estimate the evolution of the connectivity pattern during the course of learning we compared for a given pair of channels the value of dDTF averaged over all of time windows (mean dDTF) as well as the difference in dDTF values between the first and the middle time window (corresponding to SWR occurrence) (ΔdDTF) on days 1, 3 and 6 of the behavioral experiment. Since not all animals expressed SWRs that were suitable for dDTF analysis (see Material and Methods), the number of animals for which repeated measurement statistics could be performed on these days were reduced to 5 in each group. Here, for any pair of channels, neither the mean dDTF nor ΔdDTF expressed significant changes during the course of learning in either group (N = 5, p > 0.05, Friedman’s test).

### Hippocampal-cortical coupling in the SWRs frequency band

**Fig 4:**
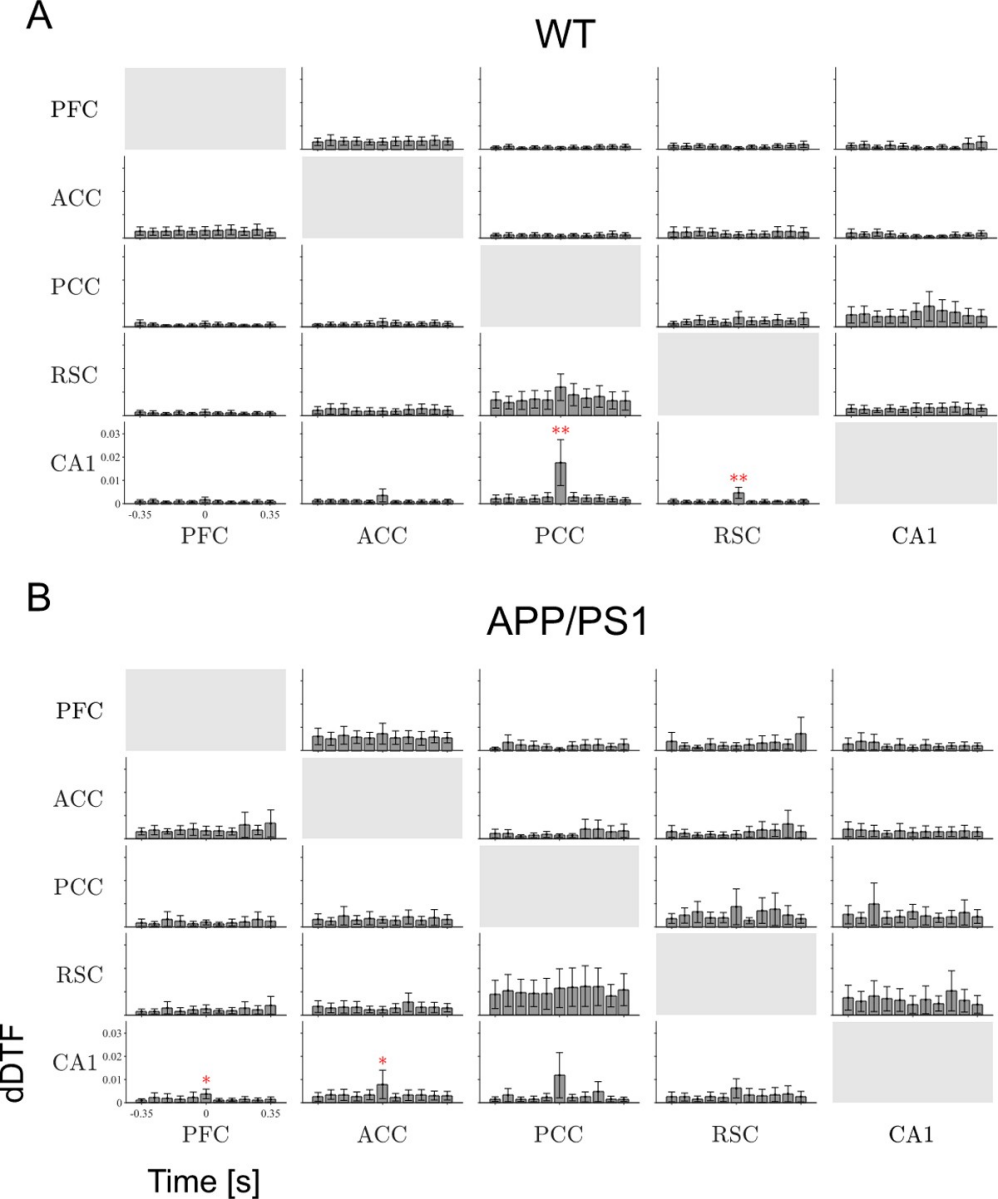
Effective coupling in the SWRs band. Time course of dDTF in SWRs (130–180 Hz) band, averaged over WT (A) and APP/PS1 (B) mice. Other abbreviations as in Figs 2 and 3. * p < 0.05, ** p < 0.01, *** p < 0.001. Time bin size is 70 ms.

**Fig 5:**
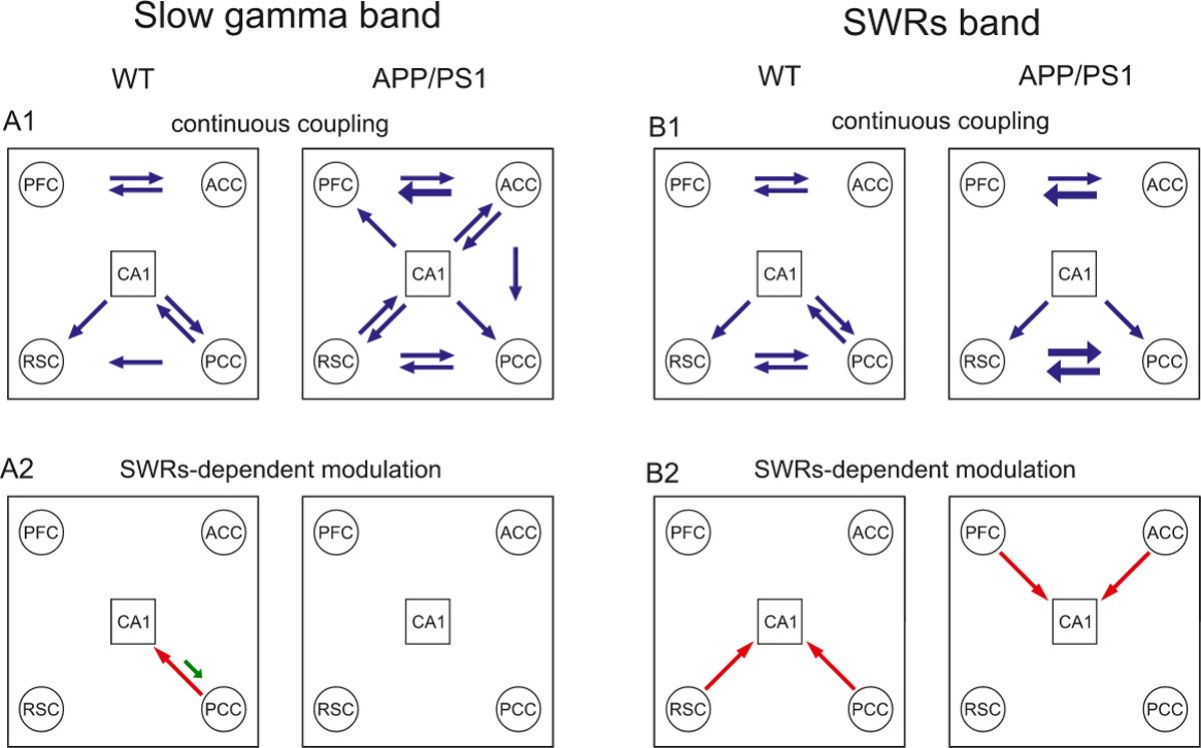
Comparison of effective coupling patterns in APP/PS1 and WT groups. The wiring diagram of effective connections between cortical areas and hippocampus (CA1) expressed in the slow gamma (A) and SWRs (B) frequency band is shown. Continuous coupling exceeding 35% of a maximal value of $\sqrt{\text{dDTF}}$ in a given group and a given frequency band are indicated by blue arrows. In the APP/PS1 group connections which significantly increased compared to the WT are indicated by thick blue arrows. A significant increase or decrease of the connectivity strength during the occurrence of SWRs is indicated by red and green arrow, respectively.

Mean values of dDTF in the SWRs frequency band, averaged over the WT and APP/PS1 groups in each of the time windows, are shown in Fig 4A and 4B, respectively, whereas a schematic diagram summarizing the most pronounced interaction is presented in Fig 5B. Interaction was not limited to the duration of SWRs but generally took place during the entire 770 ms epoch containing a SWR. In the WT group the strongest continuous coupling was identified between CA1, PCC and RSC, as well as between ACC and PFC; it was therefore similar to the pattern expressed in the slow gamma band (see Fig 4A, c.f. blues arrows, in Fig 5A1 and 5B1). Similarly, a clear-cut SWRs-specific increase of the coupling from the cortex to hippocampus was found, namely from PCC to CA1 (N = 9, p = 0.0002, Wilcoxon’s test with Bonferroni correction) and from RSC to CA1 (N = 9, p = 0.0005, Wilcoxon’s test with Bonferroni correction) (see asterisk, Fig 4A and red arrows, Fig 5B2).

In the APP/PS1 mice the pattern of continuous coupling contained similar pathways as in WT, with some inter-cortical coupling expressed more strongly (ACC to PFC: N = 9, p = 0.003, Wilcoxon’s test; RSC to PCC: N = 9, p = 0.034: RSC to PFC: N = 9, p = 0.025, Wilcoxon’s test) (Figs 4B and 5B1). Importantly, a transient increase in the effective connectivity from the cortex to hippocampus, associated with SWRs occurrence, was expressed in different cortical sites in APP/PS1 mice than in the WT group, namely from ACC to CA1 (N = 9, p = 0.004, Wilcoxon’s test with Bonferroni correction) and from PFC to CA1 (N = 9, p = 0.0019, Wilcoxon’s test with Bonferroni correction) (Figs 4B and 5B2).

Comparison of the mean dDTF as well as ΔdDTF on days 1, 3 and 6 revealed general stability of the connectivity pattern expressed in the SWRs frequency band. No significant changes were found in the connections between the majority of channels except for increasing connectivity strength from PFC to CA1 in the WT group (mean dDTF: N = 5, p = 0.041, and ΔdDTF: N = 5, p = 0.015, Friedman’s test) and from CA1 to ACC in the APP/PS1 group (ΔdDTF: N = 5, p = 0.015, Friedman’s test).

### Quantification of the expression of AB plaques

**Fig 6:**
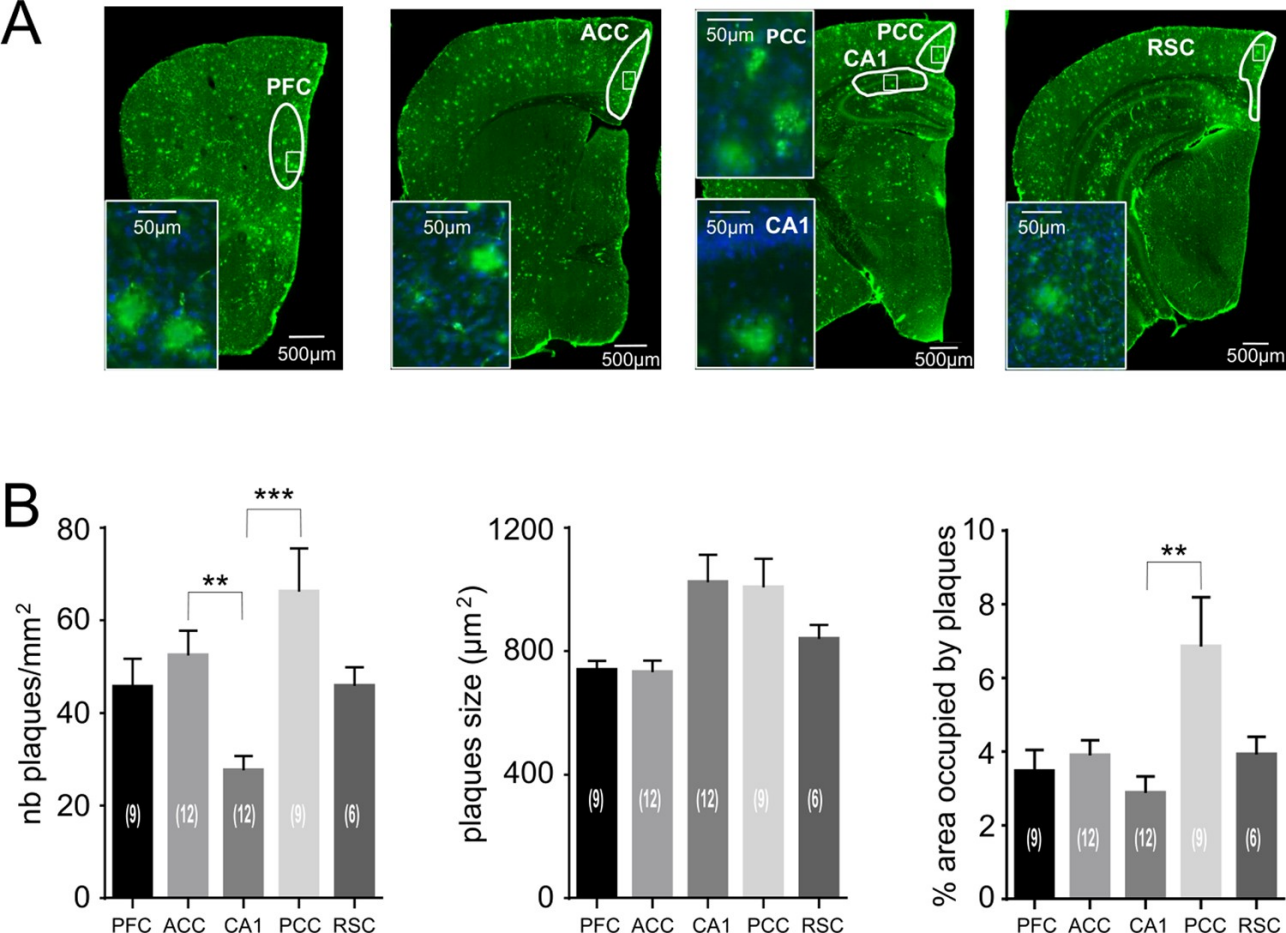
β-amyloid plaque pathology in coronal slices of APPxPS1 mice brains. β A, Anti-Aβ 1–42 immunoreactivity (6E10 + poly18058 antibodies) in coronal slices of APP/PS1 mice brains. Regions of interest are drawn in white. The inserts show higher magnification of regions delimitated by small white rectangles. Cell nuclei counterstained with Dapi (in blue). Notice that Dapi staining shows that cell nuclei are surrounding the plaques. B, Bar graphs illustrating the statistical analyses of the number of plaques (left), plaque size (middle) and area occupied by plaques (right) in the regions of interest (PFC, ACC, CA1, PCC, RSC). Notice more numerous and larger plaques in PCC versus other regions. Dunn’s test of multiple comparisons were used for statistical analyzes, ** p < 0.01, *** p < 0.001.

In order to evaluate whether the electrophysiologically-detected reconfiguration of the connectivity pattern expressed in the APP/PS1 mice correlates to morphological changes, we performed a histological study of the expression of Aβ plaques in the brain areas recorded (Fig 6). As illustrated in Fig 6A, all the coronal brain slices of APP/PS1 mice are Aβ-immunoreactive, whereas no staining was found in WT mice (data not shown). Importantly, distributions of the number of plaques expressed per mm^2^ differed significantly among the areas of interest (p = 0.001, Kruskal-Wallis test), with PCC and ACC containing more plaques per mm^2^ than CA1 (p = 0.009 and p = 0.0075, respectively, Dunn’s multiple comparisons test) (Fig 6B, left). The distributions of the size of Aβ plaques also differed among brain areas (p = 0.025, Kruskal-Wallis test) although multiple comparison testing did not show significant differences between any pair of areas (Fig 6B, middle). Finally, the comparison of the percent of the area occupied by Aβ plaques revealed significantly different distributions among the studied brain sizes (p = 0.024, Kruskal-Wallis test), with PCC being more occupied than CA1 (p = 0.012, Dunn’s multiple comparisons test) (Fig 6B, right). In sum, these results designate PCC as the region that was most affected by the Aβ pathology both in terms of the number of plaques per mm^2^ and the percentage of the studied area occupied by them.

## Discussion

We analyzed effective coupling between hippocampal and cortical circuits involved in the generation of oscillatory activity in epochs in which SWRs were generated in the hippocampus during SWS. Using dDTF method, an effective connectivity measure based on Granger causality, we showed, in both the WT and 8 to 9-month-old APP/PS1 mice, that the coupling is expressed mainly in the SWRs (130–180 Hz) and slow gamma frequency range (20–60 Hz) and undergoes alteration during the occurrence of SWRs. The pattern of connectivity and its SWR-dependent dynamics were different in APP/PS1 and WT groups.

In the WT group the hippocampal-cortical interaction in both frequency bands was identified between CA1, PCC and RSC with the additional reciprocal connections between ACC and PFC (see blue arrows, left panels, Fig 5A1–5B1). In APP/PS1 mice, a similar coupling pattern was found in the SWRs frequency band (see blue arrows, right panel, Fig 5B1) with some connections becoming stronger than in the WT group (see thicker blue arrows, right panel, Fig 5B1). However, in the slow gamma band, the connectivity pattern found in the APP/PS1 animals was much more wide-spread than in the WT group. Namely, new intra-cortical couplings as well as the interaction between hippocampus and all cortical areas studied were robustly expressed (blue arrows, c.f. right and left panel, Fig 5A1).

Interestingly, effective coupling was modified during occurrence of SWRs differently in both groups. In the slow gamma band, in the WT group, the strength of connectivity from PCC to CA1 increased during generation of SWRs, whereas the connectivity strength in the reverse direction, from the CA1 to PCC, decreased (see, red and green arrow respectively, left panel, Fig 5A2). By contrast, in the APP/PS1 group no changes related to SWRs occurrence were expressed (right panel, Fig 5A2). In the ripple band, during SWRs, the connectivity strength from the cortex to hippocampus increased in both groups, however involving different cortical areas: from PCC and RSC to CA1 in the WT, and from ACC and PFC to CA1 in the APP/PS1 mice (Fig 5B2).

Altogether these data indicate that patterns of interactions between hippocampus and multiple cortical sites, expressed in the slow gamma and ripple frequency band, were altered in an advanced neurodegenerative state in the APP/PS1 mice. However, APP/PS1 mice expressed the same reference memory performance as the WT group [22], therefore this alteration may be considered an adaptive reconfiguration of cortical-hippocampal network putatively allowing memory formation despite neurodegenerative process.

APP/PS1 mouse is a model of amyloidosis, however it also reproduces cognitive impairment as well as anxiety, hyperactivity and social interaction impairment [26]. Moreover, Tau hyper-phosphorylation and phosphorylated Tau-positive neuritic processes have been observed in the vicinity of amyloid deposits [31, Macrez; unpublished results]. Amyloidosis and cognitive impairment occur early (5–9 months old) in this double transgenic AD mouse model, but Tau aggregation as tangles and neuronal loss are missing in this model. Whether absence of Tau tangles can account for the ability to reconfigure the pattern of effective coupling between the hippocampus and cortex remains to be explored in other models.

Functional connectivity has been studied in anesthetized APP/PS1 mice using an optical method assessing correlations in activity of various brain regions based on oxygenated blood flow signal within the Default Mode Network [32]. They showed that functional connectivity is decreased in aged mice in regions where Aβ plaques are most present, whereas our data showed an increase of connectivity strength in APP/PS1 mice compared to the WT in the slow gamma frequency (Fig 5A). However, the method used and the definition of functional connectivity differs substantially from that adopted by our group.

The method used in our study originates from an idea of causal relations that has been developed by Granger [33] and Wiener [34]. They proposed that, for two simultaneously measured time series, one time series can be called causal to the other if we can better predict the second time series by incorporating knowledge about the first one. dDTF, an extension of Granger causality principle to a multichannel case, is an estimator of *effective* connectivity, which implies causal coupling between time series (see Materials and Methods), whereas *functional* connectivity (calculated, e.g., by means of correlations or coherence) indicates only statistical interrelation between signals.

Effective coupling between cortical and hippocampal networks in the ripple frequency band was expressed both outside SWRs and during SWR occurrence (Figs 4 and 5B1). The former indicates coupling between cortical signals and CA1 signals which were of low amplitude (< 5 SD) or long duration (> 100 ms) and therefore not identified as SWRs (see Materials and Methods). Surprisingly, in both groups, this coupling increased during occurrence of SWRs only in one direction, from the cortex to hippocampus, despite the fact that SWRs are generated in the hippocampus. This suggests that cortical networks may promote SWRs incidence and thereby initiate a transfer of information, presumably encoded and expressed in a time-compressed form in SWRs, from hippocampus to cortex. Indeed, ripple-like oscillations were found recently in multiple cortical areas and their involvement in spatial learning was demonstrated [35] [Khodagholy et al. 2017]. Interestingly, we have shown recently that in WT animals SWRs were often temporally associated (co-occurring) with PCC and RSC ripples whereas in APP/PS1 mice the co-occurrence of SWRs with ACC and PFC ripples was higher than in WT [22]. This finding is in agreement with the changes in coupling expressed in the ripple frequency band during SWRs occurrence, where the coupling from PCC and RSC to CA1 in the WT mice is replaced by the coupling from ACC and PFC to CA1 in APP/PS1 mice (see Fig 5B2); this further supports the idea of cortical-hippocampal ripple-to-ripple interaction.

As we recorded from the dorsal part of hippocampus, which has connections with the posterior part of cingulate cortex [13], the effective cortical-hippocampal coupling in which mainly PCC activity targets CA1 in the WT group (Fig 5A2 and 5B2) may reflect an actual anatomical connectivity. In the APP/PS1 group the effective coupling between cortex and hippocampus involves the anterior and not the posterior part of cingulate cortex (Fig 5B2), which may be a sign of a more advanced neurodegenerative stage of PCC. This possibility is further supported by results of the histological analysis, indicating PCC as the region of the most advanced Aβ pathology among the brain areas considered in the present study (Fig 6B).

It has been recently shown, in a study of brain effective coupling estimated by resting state eyes-closed EEG, that DTF values were lower in a group of AD patients than in a group of normal elderly subjects, although the connectivity pattern was similar in both groups [36]. These results are quite different from what we found in the APP/PS1 model of AD. Although such translational conclusions are always very difficult, one can imagine that the neurodegenerative process was more advanced in this human study than in our animal group. Indeed, neurodegeneration in APP/PS1 mice starts at 5 months and develops progressively until 12 months in females. From this perspective, the 8-month old animals may still be in the middle of the neurodegenerative process when the brain can still express some plasticity, which may allow for reconfiguration of cortical-hippocampal interaction and provide temporally adaptive compensatory mechanisms permitting memory formation—an ability that may disappear in an advanced stage of the expression of the illness. Since the method of estimation of effective connectivity has been used successfully to distinguish AD patients from an elderly healthy people group [36] it would be interesting to apply dDTF estimation of brain connectivity pattern as an early marker of AD.

Our results indicate that the SWR-related effective coupling between hippocampus and cortex occurs in two frequency bands, one of which is slow gamma oscillations (20 to 60 Hz) (Fig 2). This later coupling is even stronger in APP/PS1 animals compared to the WT group (Fig 5A1). Interestingly, it has been shown that the optogenetically driven gamma oscillation at 40 Hz reduced levels of cognitive decline in an AD mouse model [37]. This rises a possibility that an enhanced cortical-hippocampal interaction in the slow gamma band expressed in APP/PS1 mice may contribute to the ability of this AD model to perform a reference memory task at the same level as the WT group [22].

In conclusion, our data show reorganization of the cortico-hippocampal effective coupling pattern in epochs containing SWRs in APP/PS1 mice in a middle neurodegenerative state. Moreover, the results indicate the direction of the causal coupling as from the cortex to hippocampus in both WT and APP/PS1 groups during the occurrence of SWRs and the possible involvement of cortico-hippocampal ripple-to-ripple dialogue in the information transfer.
